# Investigating the effects of pelagic trawling on the welfare of Atlantic herring (*Clupea harengus*)

**DOI:** 10.1038/s41598-024-68629-8

**Published:** 2024-07-30

**Authors:** Mafalda Tomás, Jane W. Behrens, Dennis Brandborg Nielsen, Claus Reedtz Sparrevohn, Manuel Gesto, Fintan McEvoy, Albin Gräns

**Affiliations:** 1 Department of Applied Animal Science and Welfare, Swedish University of Agricultural Sciences, Gothenburg, Sweden; 2 DTU Aqua-National Institute of Aquatic Resources, Technical University of Denmark, Kongens Lyngby, Denmark; 3 Division of Food and Production, Danish Technological Institute, Taastrup, Denmark; 4 Danish Pelagic Producers Organisation, Copenhagen, Denmark; 5 Department of Veterinary Clinical Sciences, University of Copenhagen, Frederiksberg C, Denmark

**Keywords:** Welfare, Reflex impairment, *Clupea harengus*, Trawl, Pelagic fisheries, Animal physiology, Ichthyology

## Abstract

The effects of pelagic trawling on the health and welfare of Atlantic herring (*Clupea harengus L.*) were investigated on a refrigerated seawater vessel operating in the North Sea. A total of 495 Atlantic herring (*Clupea harengus L.*) were sampled during five hauls from two fishing trips in September 2021 and 2022. For assessments of consciousness and mortality, a Reflex Action Mortality Predictor test (*i.e*. RAMP-test) was used on herring collected following trawling and pumping. Inspections for external and internal damage or wounds were performed via morphological welfare indicators and analyses of photos and radiographs. In addition, blood samples were taken and analysed for haematological indicators of stress. Following trawling and pumping, only 5% of the investigated herring showed signs of external wounds associated with the morphological indicators of welfare, and no internal damage was observed in the radiographic inspections. However, 96% of the assessed herring scored 0 on all three reflexes included in the RAMP-test and were therefore judged dead. On average, herring lost 95% of their scales, while 95% of herring had a very high degree of ruptured red blood cells (*i.e.* haemolysis). Extensive scale loss results in a deterioration of the skin's protective barrier function, which in turn impairs the osmoregulatory capacity of the herring. This was evident by elevated levels of plasma osmolality and circulating chloride concentrations, which could also likely explain the high occurrence of haemolysis in captured herring. Extended trawling time and larger catch size proved to be two important factors to consider, as the former led to increased plasma levels of osmolality, whereas the latter was associated with elevated plasma levels of lactate and cortisol. In conclusion, the high mortality appears to be influenced by a combination of factors such as severe stress, loss of osmoregulatory ability, crowding density within the trawl, and extended trawling times. This study provides important information on the welfare of wild Atlantic herring caught using pelagic trawls and highlights areas where improvements can be made to safeguard the welfare of fish captured in pelagic fisheries in the future.

## Introduction

Fish caught from wild stocks constitute the primary source of animal protein for a large proportion of the ever-growing human population^1^, and more than 90 million tonnes of fish are caught in commercial fisheries globally every year^1–3^. Thus, both small and large-scale fisheries are socio-economically important, especially in coastal areas^4–6^. To mitigate overfishing or depleting fish stocks, various management measures such as fishing quotas and protected areas are constantly implemented and adjusted^4,7–9^. A much less discussed aspect of commercial fishing is one that considers the animal welfare of captured individuals. Here, we use the definition of “animal welfare” proposed by the World Animal Health Organization (WOAH): “Animal welfare means the physical and mental state of an animal in relation to the conditions in which it lives and dies”. Furthermore, a good state of animal welfare, in relation to capture and killing, is one where an animal is not suffering from unpleasant states such as pain, injury, fear, and distress.

Approximately 68% of fish caught by humans are done so in pelagic fisheries^1,2,5,6,10^. Within the Danish pelagic fisheries alone, around 30 billion fish are estimated to be caught from the wild each year, making it, by numbers, the largest in the EU^3^. Pelagic fisheries often target small and some less familiar species, and their catches are mainly intended for fish oil and fish meal production instead of human consumption^11^. Therefore, little attention has been given to the animal welfare aspects of fish caught in pelagic fisheries. However, with growing evidence regarding the sentience of fish, the concern and awareness about fish welfare problems have increased among consumers, producers, non-governmental organisations (NGOs) and authorities alike^12^.

One potentially significant fish welfare problem concerns the high mortalities observed in pelagic fisheries compared to other fishing methods, such as traps, gillnets, and hooks^13^. Pelagic fishermen report that the quantity of fish appearing dead after being pumped onto the fishing vessel can be extremely variable (personal communication with the crew on board HG62 Beinur). The underlying reasons for this substantial variation and, ultimately, how, why, and where fish die during fishing operations, remain unclear. However, wounds and other skin injuries, particularly scale loss, have been identified as some of the most critical factors affecting fish survival^14–17^. Additionally, trawling time^18,19^, catch size^17,19–24^, catch depth^15,25^ and water temperature^19,20,26^ have also been identified as potentially critical. Today, efforts are made to develop fishing gear and routines that minimise handling and are gentler on the fish, as excessive stress prior to death will have negative effects on both fish welfare and product quality^27–31^.

Examples of some efforts to improve fish welfare primarily involve gear modifications, such as variations in mesh thickness and size^32^, the use of selective devices, different materials, and modifications to codend construction and materials, as well as alternative mesh configurations^33^. Additionally, hydro-acoustic technology is employed to estimate biomass and identify species^34^, and slipping practices are being developed to safely release catches from the net while still in the water, minimising mortality risk^34,35^. However, these developmental efforts are largely constrained by the lack of knowledge regarding just how stressful different fishing gear and routines are to the fish and how the physiological stress response relates to fish welfare during commercial fishing.

Fishing methods and routines that minimise stress and are gentle on the fish can also be problematic from a fish welfare perspective, as fish caught by pelagic fisheries are usually not actively killed on board the vessel. For example, many larger pelagic trawl vessels pump captured fish from the ocean into tanks filled with refrigerated seawater (RSW), where they remain until further processing ashore^10,24^. It has been noted that in some cases, fish may even remain alive upon arrival at processing factories, many hours post-capture (personal communication with the crew on board of HG62 Beinur). Consequently, neither dying on board the vessel nor in the fishing gear is likely to be a humane death (*i.e.* one without experiences of fear, anxiety, pain and distress).

One species commonly caught in large numbers using pelagic trawls is the Atlantic herring (*Clupea harengus L.*), and in recent times, as many as 20 billion herring have been caught annually in the North Atlantic Ocean^36^. In addition, Atlantic herring is an essential element of the North Atlantic's marine ecosystem, being a critical species in the food web^37^. Therefore, Atlantic herring is, both today and historically, a highly ecologically, nutritionally and financially important species for the Nordic countries^37–39^. With the high number of Atlantic herring being caught in the pelagic trawling fishery, any means, which can work towards safeguarding their welfare during capture and killing are highly desirable from an animal welfare perspective.

In the present study, we aimed to assess the welfare of Atlantic herring (*Clupea harengus L.*) after capture with pelagic trawling. To achieve this, we had an opportunity to join a commercial RSW vessel trawling for Atlantic herring in the North Sea for two consecutive years. We assessed the health and welfare of Atlantic herring following pelagic trawling and pumping onto the vessel using i) indicators of consciousness/mortality, such as equilibrium, rhythmic opercular activity and response to tactile stimuli, ii) morphological welfare indicators, such as barotrauma, fin damage, skin lesion, operculum damage and eye damage and iii) haematological indicators of stress, such as plasma cortisol concentration, plasma lactate, total plasma osmolality and plasma chloride. As catch size and trawling time have been highlighted as two potential critical factors affecting the health and welfare of fish caught in pelagic fisheries^19^, we hypothesised that an increase in catch size and trawling time would have detrimental impacts on the health and welfare of herring. To better understand the severity of the stress experienced by the Atlantic herring following trawling, all haematological indicators of stress were compared with a reference group of fish caught using the relatively quick hook and line method (see Fish collection for more details). We anticipated that the more intense nature of trawling, characterised by the longer durations and the process of chasing fish until exhaustion, would result in significantly higher stress levels compared to the relatively quick hook-and-line method^40,41^. Finally, given that trawling operations on a commercial RSW vessel can extend over several hours, resulting in a significant proportion of fish being dead when pumped onto the vessel, we investigated the potential impact of post-mortem intervals on haematological stress indicators. Given that fish caught by trawling are subjected to exhaustive chasing and tow durations that can last for several hours, it is reasonable to assume these factors would result in higher concentrations of these indicators^19^.

## Material and methods

### Fish collection

A total of 495 Atlantic herring (*Clupea harengus L.*), with fork lengths of 28.94 ± 1.83 cm (mean ± standard deviation), were assessed following capture during five hauls from two fishing trips in September 2021 and 2022 (Table 1). These fish were caught using a 500 m trawl with a novel T90 codend (as shown in Fig. 1 in the supplementary material) with an opening of approximately 100 m, hauled at a speed of 3–3.5 knots by a 78 m RSW vessel (with a total holding capacity of 2600 m^3^) in the North Sea. Following trawling, the fish were pumped from the net to the holding tank, and then sampled at the water separator where they were subjected to mechanical sorting. All individuals were randomly sampled throughout the pumping process, which took approximately 30 min. During the entire process, fish were subjected to various mechanical stressors, including the net’s motion and hydraulic pressures, which exert forces such as pressure and fracture.

**Table 1:** Summary of the five hauls that are included in the study.

Haul	Date	Number of individuals	Trawling time	Catching depth	Catch size	Pump velocity
1	18 September 2021	60	120 min	145–160 m	200 tonnes	20–25 t m^−1^
2	18 September 2021	40	300 min	145–160 m	350 tonnes	20–25 t m^−1^
3	19 September 2021	130	180 min	145–160 m	280 tonnes	20–25 t m^−1^
4	8 September 2022	40	60 min	60 m	690 tonnes	15–20 t m^−1^
5	8 September 2022	224	60 min	60 m	560 tonnes	15–20 t m^−1^

A pelagic trawl was used to capture Atlantic herring (*Clupea harengus*) in the North Sea.

Since the above mentioned herring are likely subjected to a series of potentially stressful and harmful events during pelagic trawling (*e.g.* capture, crowding in the trawl, exhaustion, decompression, asphyxia and mechanical damage from the trawl and the pump^26,31,42^, it is necessary to obtain reference values for the measured indicators described in subsequent sections from ‘resting’ or ‘unstressed’ herring. This was achieved by capturing 40 Atlantic herring (here after referred to as ‘reference fish’) with fork lengths of 25.75 ± 2.66 cm using hook and line in Øresund on November 23^rd^, 2021.

Hook and line is one of the fastest methods of capturing herring, and the hauling time in the present study was approximately 30 s. This method allows for rapid attainment of specific depths, often within minutes, facilitating the retrieval of fish, typically accomplished within a five-minute timeframe per catch. In contrast, trawling necessitates the use of larger nets, resulting in a prolonged process of 15 to 30 min to reach desired depths, followed by several hours of trawling at speeds ranging between 2–4 knots and additional time required for retrieval^40,43^. It has previously been documented that many hormonal and ionic stress-related blood parameters remain within resting levels if blood samples are obtained quickly enough after capture^44^. Thus, these fish were collected as quickly as possible, euthanised with a blow to the head by a fish priest, and immediately sampled using the methods described below.

### Investigated variables and sample analyses

The health and welfare of Atlantic herring from different experimental groups (as outlined below) were investigated using indicators of consciousness and mortality (equilibrium, rhythmic opercular activity and response to tactile stimuli), morphological welfare indicators (barotrauma, fin damage, skin lesion, operculum damage and eye damage) and haematological indicators of stress (plasma cortisol concentration, plasma lactate, total plasma osmolality and plasma chloride). Responsive fish were collected and put into buckets to better assess the consciousness indicators.

#### Assessments of consciousness and mortality

Assessments of consciousness and mortality were made by applying the following three reflex action mortality predictors (*i.e*. a RAMP-test):

1. Equilibrium, determine whether the individual can return to their upright position when orientated upside down.
2. Rhythmic opercular activity (ROA), determine whether the individual displayed any rhythmic opercular movements.
3. Response to tactile stimuli (RTS), determine whether the individual displayed any reactions when pinched in the caudal area.

Each individual's responses were scored as 0 (absence of response) or 1 (positive response). When fish scored 0 on all three reflexes, they were presumed dead^18,19,45^. All interpretations of visual indicators of consciousness (e.g., when using the RAMP test) must be treated with caution. Visual indicators such as eye rolling or ventilation can significantly underestimate the time it takes for a fish to lose consciousness, as these signs can disappear long before neurophysiological indicators of consciousness are lost^46^. However, given the impracticality of assessing consciousness using neurophysiological methods aboard the fishing vessel, we opted to employ the RAMP test, which is both practical and widely utilized for such investigations^18,19,45^.

#### Morphological welfare indicators

A range of morphological welfare assessments were performed immediately following capture using the following five indicators^13,45^:

1. Barotrauma, determine whether there are any signs of bulging in the eyes and swim bladder (since this assessment was solely based on the external appearance of the fish, only buoyancy regulation and the expanded swim bladder, which causes the stomach to protrude from the fish's mouth, were evaluated).
2. Fin damage, determine whether there are signs of fin loss or haemorrhages.
3. Skin lesion, determine whether any wounds are leading to an exposed muscle.
4. Operculum damage, determine whether there are any shortened or missing opercula leading to exposed gill tissue.
5. Eye damage, determine whether there are any typical signs of mechanical trauma such as haemorrhages and ruptured eyes.

Morphological welfare indicators were assessed using a three-point categorical scale: 0 (no evidence of damage), 0.5 (moderate damage), and 1 (severe damage)^45^. The initial classification was performed on-site by a single evaluator and later verified in the laboratory using the photographs taken during the assessment.

The percentage of scale loss and occurrence of internal damage were also investigated in detail using image analyses and radiography. To determine the percentage of scale loss, individuals were photographed in a custom-made photo box (25 cm L × 10 cm W × 32 cm H) using a webcam with an 8-megapixel resolution (HD Webcam C615, Logitech, USA). All digital pictures were stored and later downloaded and analysed using ImageJ (National Institutes of Health, Bethesda, MD, USA) to calculate the proportion of scale coverage. Only the right side of the fish was assessed (dorsal and ventral side).

The occurrence of internal damage and deformities following trawling and pumping was also investigated. Fish were individually bagged, ID-tagged and frozen in a -20 °C freezer on-board the vessel. When returning to the university (Danish Technological Institute [DTI] in collaboration with the Department of Veterinary Clinical Sciences at the University of Copenhagen), radiographs were obtained from each fish using a clinical digital radiography system (Fujifilm DR-ID 300CL APL Software V8.0, Fujifilm Europe, Duesseldorf, Germany). Fish were placed on their side and flattened against the detector plate for exposure without using an anti-scatter grid. Radiographic exposure factors were 50 KVp and 4 mAs using a detector focus distance of 1 m. Images from the detector plate were processed using the system software and transferred to a patient picture archiving and communication system (PACS) for later evaluation. Each radiograph was inspected for signs of spinal fractures or other types of internal injuries, such as compressions, emphysemata and fluid accumulations. Radiographs were scored as 0 (no evidence of damage) or 1 (evidence of spinal fracture/internal injuries).

#### Haematological indicators of stress

To avoid the effects of handling stress, all individuals sampled at the fish/water separator (when captured during pelagic trawling) or when hauled onto shore (when captured with hook and line) were quickly euthanised with a sharp blow to the head using a fish priest. Blood samples were immediately collected from the caudal vein using a BD Microlance TM 3 23G needle with 1 ml heparinised syringes from herring captured by pelagic trawling (N = 193) and hook and line (N = 40). During pelagic trawling, more than half of the samples (N = 130) were obtained from randomly selected individuals from Hauls 1, 2, and 4. The remaining samples (N = 63) were collected from targeted individuals in Hauls 3 and 5. The rationale for selecting individuals was based on the observation that most randomly sampled individuals exhibited haemolysis, a condition where red blood cells rupture and release their contents into the blood plasma. In contrast, haemolysis was not observed in the targeted responsive fish.

Blood samples were centrifuged at 2000 g for at least 10 min (VWR MiniStar) to separate the plasma, which was subsequently pipetted out and stored at -20 °C on the vessel. Upon return to shore, plasma samples were stored at -80 °C for further analyses of plasma cortisol, lactate, osmolality, and chloride. Plasma cortisol concentration was determined by an enzyme-linked immunosorbent assay (ELISA) (ref. 042,710; Neogen Europe, Ayrshire, Scotland, UK). Plasma lactate was analysed using Sigma's colourimetric test (ref. MAKO64; St. Louis, MO, USA) following manufacturer instructions. Total plasma osmolality was estimated using a vapour pressure osmometer (Vapor Model 5600, South Logan, UT, USA) after following the calibration procedure with Opti-Mole ampule osmolality standards (OA-100, OA- 029, and OA-010) (ref: oa-100; oa-029; oa-010, South Logan, UT, USA). Plasma chloride was measured using a chloride analyser (Sherwood M926, Cambridge, UK).

### Research questions, experimental groups and statistical analysis

Getting access to fish directly after capture with a large commercial RSW vessel is challenging and, hence, rarely done. Therefore, to make the most of the present opportunity, the fish were divided into different experimental groups in order to give answers to a suite of research questions, as outlined below. All data was analysed using the statistical software R, version 4.1.2 (http://www.R-project.org). A Shapiro-Wilkins normality test was used to assess normal distribution, and a Levene's test was performed to check for equal variance. Nonparametric tests were used if the data did not meet the normality assumption and equal variance. Statistically significant results were assessed with a p-value of < 0.05.

#### The effects of trawling time and catch size on fish welfare

In this experimental group we aimed to investigate the effects of trawling time and catch size on fish welfare. To achieve this, we investigated the percent scale loss and plasma concentrations of cortisol, lactate, osmolality, and chloride in herring actively targeted at the separator, as they exhibited clear signs of consciousness in the RAMP test (N = 63). These parameters were examined at trawling durations of 60 and180minutes and catch sizes of 280 and 560 tonnes (Hauls 3 and 5). Morphological indicators (n = 40) and assessments of consciousness and mortality (n = 40) were conducted on randomly selected fish at trawling durations of 60, 120, and 300 min, and at catch sizes of 200, 350 and 690 tonnes (Hauls 1, 2 and 4). In addition, and for reference purposes, all variables were compared with the reference fish caught using hook and line (N = 40). Here, the effect of trawling time and catch size on the different variables were investigated using a Kruskal Wallis rank-sum, followed by a Wilcoxon rank-sum test for pairwise comparisons if significant differences were detected. All p-values were adjusted following the Bonferroni correction. Correlations between all the variables were made using a Spearman correlation test.

#### The relationship between haemolysis and moribund fish

When inspecting the plasma samples from Atlantic herring caught with the trawl, it was evident that a high proportion of the blood samples were haemolysed. This phenomenon has been documented in a study on Atlantic herring, highlighting how their physiological responses and mortality are influenced by scale loss^47^. Therefore, in order to investigate whether haemolysis is more prevalent in dead fish, we compared the occurrence of haemolysis in herring (n = 40) randomly collected (Haul 1, 2 and 4) from the fish/water separator of the vessel (*i.e.* immediately after being pumped out of the trawl) with herring that were actively targeted (Haul 3 and 5) at the separator as they showed signs of consciousness in the RAMP-test. In addition, the occurrence of haemolysis in dead fish compared to targeted live fish was investigated using Pearson's Chi-squared Test for count data.

#### The effect of time elapsed since death

As the trawling on a commercial RSW vessel can go on for several hours, and a high ratio of fish are potentially already dead when pumped onto the fishing vessel, it is inevitable that sampled individuals experienced different trawling times and were sampled at different times post-mortem. Thus, to accurately quantify the effect of time elapsed since death, we examined temporal changes in the investigated haematological variables in herring that were captured using hook and line (n = 20). All individuals received full scores in the assessment of consciousness and were euthanised in buckets filled with 12L of non-aerated seawater and tricaine methanesulphonate (MS-222, 150 mg/L) buffered with NaHCO_3_ (300 mg/L). These fish were then sampled for blood randomly after 15, 30, 45, 60, 120 and 180 min, respectively. Blood samples were centrifuged at 2000 g for 10 min (VWR MiniStar) to separate the plasma, which was subsequently pipetted out and stored on the vessel. Here, the effect of time elapsed since death on plasma levels of cortisol, lactate, osmolality and chloride was investigated using linear regression.

## Results

### General effects of trawling and pumping

**Figure 1:**
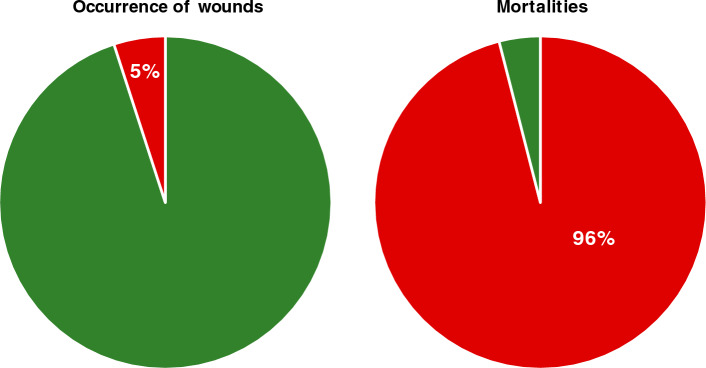
Disparity in the investigated indicators of welfare. Following trawling and pumping only 5 % of all sampled Atlantic herring (Clupea harengus) that were assessed had wounds on their skin, eyes or fins, whereas 96 % were presumed dead as they scored 0 on all three reflexes in the RAMP.

Following trawling and pumping, only 4% (19 individuals) of the assessed fish exhibited signs of reflex responses (Fig. 1). Among these fish, 26.3, 52.6, and 57.9% showed signs of equilibrium, rhythmic opercular activity and response to tactile stimuli, respectively. This largely contrasts with the reference fish (caught using hook and line), where 100% of the sampled fish scored 1 on all the reflex responses.

**Figure 2:**
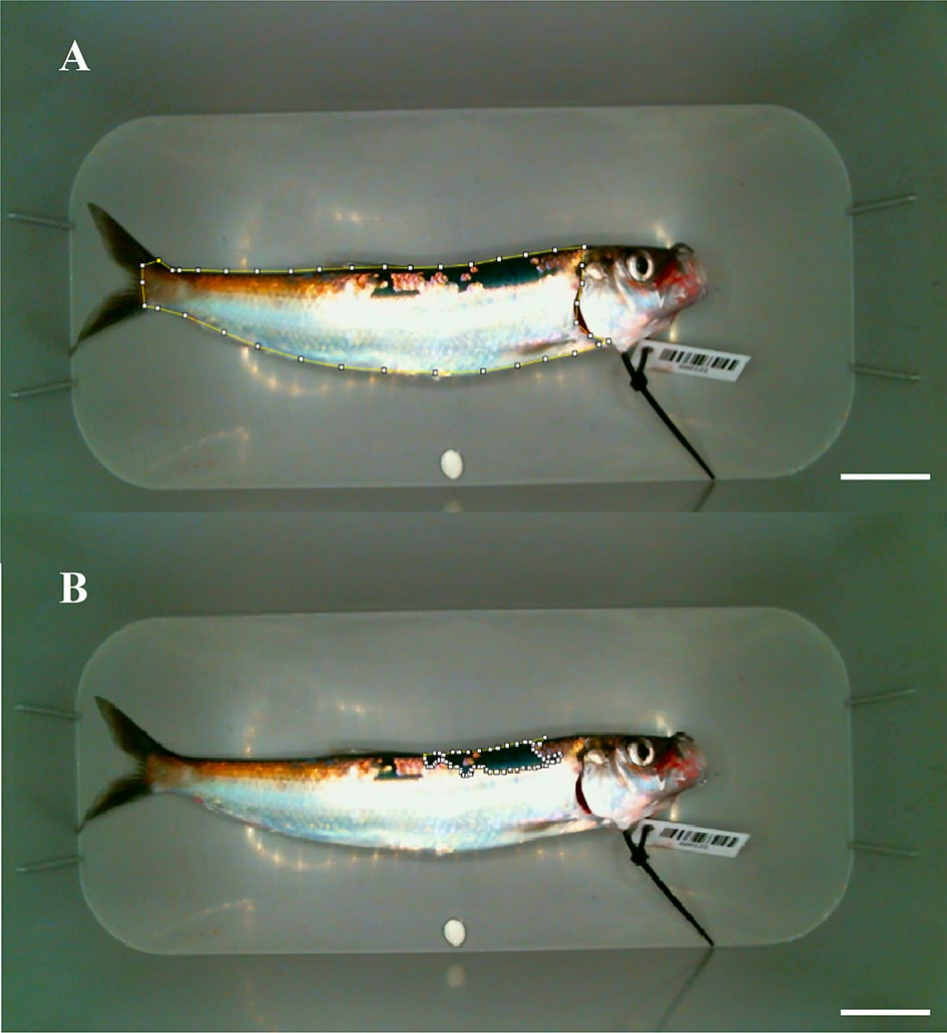
Determination of the percentage of scale loss. Photos of Atlantic herring (*Clupea harengus*) taken with a custom-made photo-box were analysed with ImageJ using the polygon and measure tool. The scale coverage in percentage was calculated from one side of the fish as the proportion between the total area of scales (here marked in A) and the areas where scales were missing (one such area marked in B). The white bars represent a reference scales of 4.8 cm.

**Figure 3:**
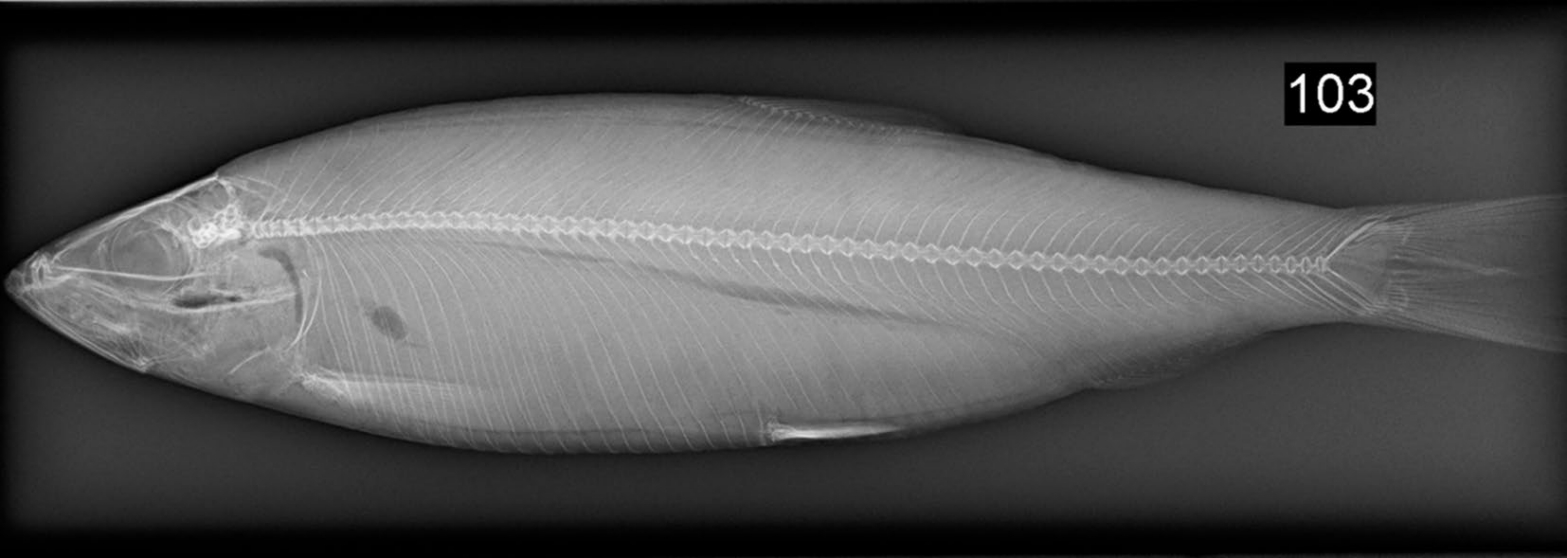
Radiograph from an Atlantic herring (*Clupea harengus*) used for visual inspections for internal trauma and deformities following trawling and pumping. No signs of spinal fracture or any other types of internal injuries (e.g., compressions, emphysemata and fluid accumulations) were found on any of the inspected individuals.

In addition, fish caught with the trawl experienced extensive scale losses (Fig. 2). On average, trawled individuals lost approx. 95% of their scales. Despite the excessive scale loss, only 5% (23 individuals) of the sampled fish displayed other morphological indicators of poor welfare (Fig. 1), such as pressure damage, fin damage, skin lesions, opercular damage, and eye damage. None of the sampled fish showed signs of internal trauma or deformities on their radiographs (Fig. 3). Out of the observed morphological indicators, eye damage was the most prevalent, occurring in 82.6% of the wounded fish. In comparison, fin damage and opercular damage were observed in 13.0 and 17.4% of wounded fish, respectively. No sampled fish showed signs of pressure damage or skin lesions.

Trawling followed by pumping also triggered a pronounced stress response in the plasma levels of the investigated haematological stress indicators. Plasma levels of cortisol, lactate, osmolality, and chloride were all significantly higher than the reference fish (Table 2). The most pronounced effect was seen in the circulating concentrations of the stress hormone cortisol, where trawled fish, on average, had almost 500 times higher levels than the reference fish (0.4 ± 1.4 ng/mL compared to 192.6 ± 172.4 ng/mL of trawled and reference fish, respectively).

**Table 2:** Summary of sampled blood hematological parameters in Atlantic herring (*Clupea harengus*) caught with a pelagic trawl (trawling) or with hook and line (reference).

	Cortisol (ng ml^−1^)	Lactate (mM)	Osmolality (mmol kg^−1^)	Chloride (mmol l^−1^)
Trawling	192.6 ± 172.4^a^	21.1 ± 10.3^a^	484.3 ± 51.8^a^	185.5 ± 30.2^a^
Reference	0.4 ± 1.4^b^	1.0 ± 0.5^b^	347.2 5 ± 28.2^b^	140.2 ± 16.5^b^

Dissimilar letters represent significant differences between groups (p<0.05) comparison was made with a Kruskal Wallis rank-sum test.

Values are presented as average ± S.D.

### The effect of trawling time & catch size on fish welfare

**Figure 4:**
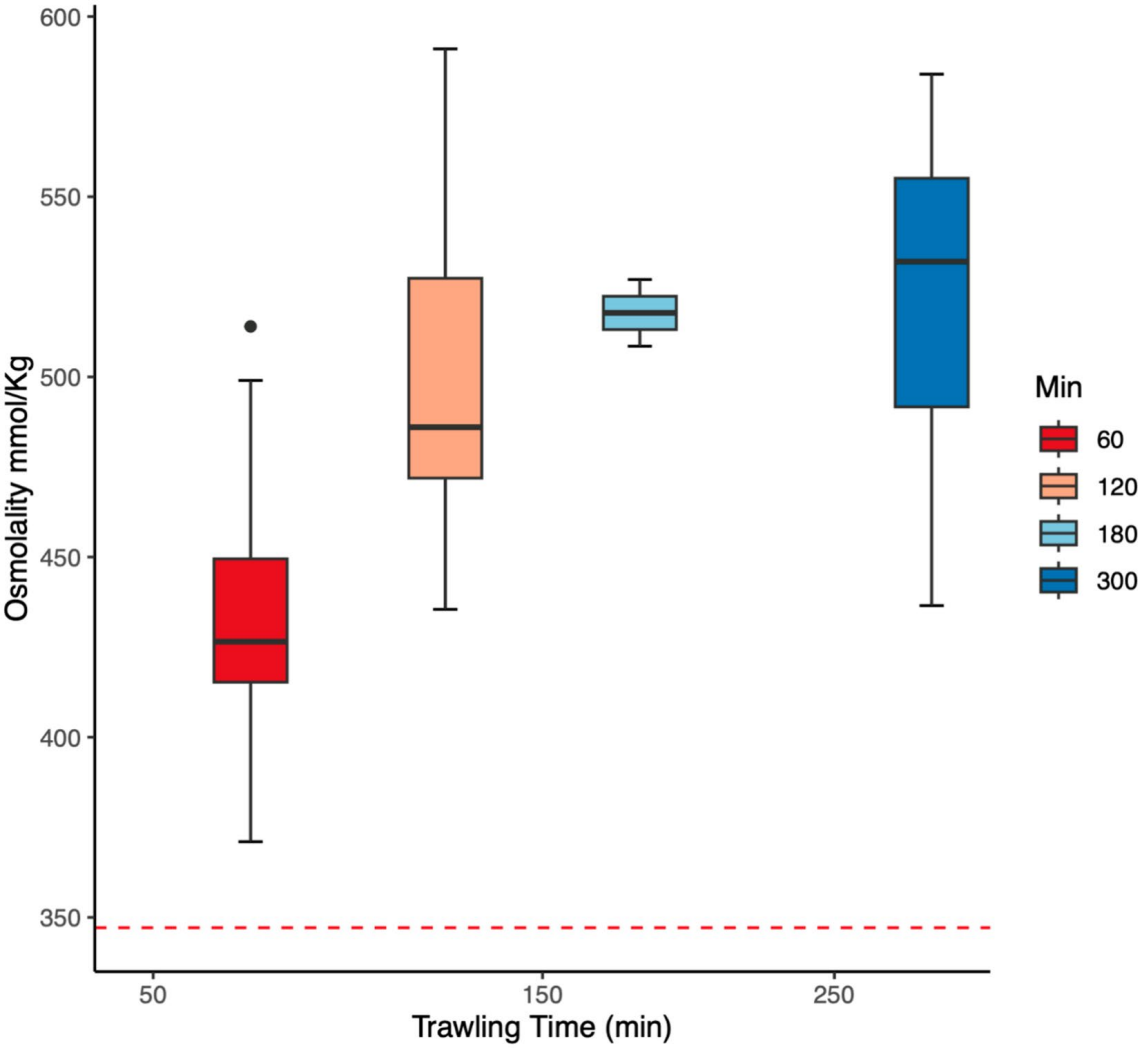
Plasma osmolality mmol kg^−1^. of Atlantic herring (*Clupea harengus*) following trawling and pumping in the north sea at four different trawling durations*. *Values at each trawling time are presented as box-plots and the outcome of a Spearman correlation test showed a moderate (correlation coefficient of 0.50) and significant (p<0.001) positive relationship between trawling time and plasma osmolality. The dashed red line is included for illustrative purposes only and represents the mean plasma osmolality in the group of reference fish caught with a hook and line.

**Figure 5:**
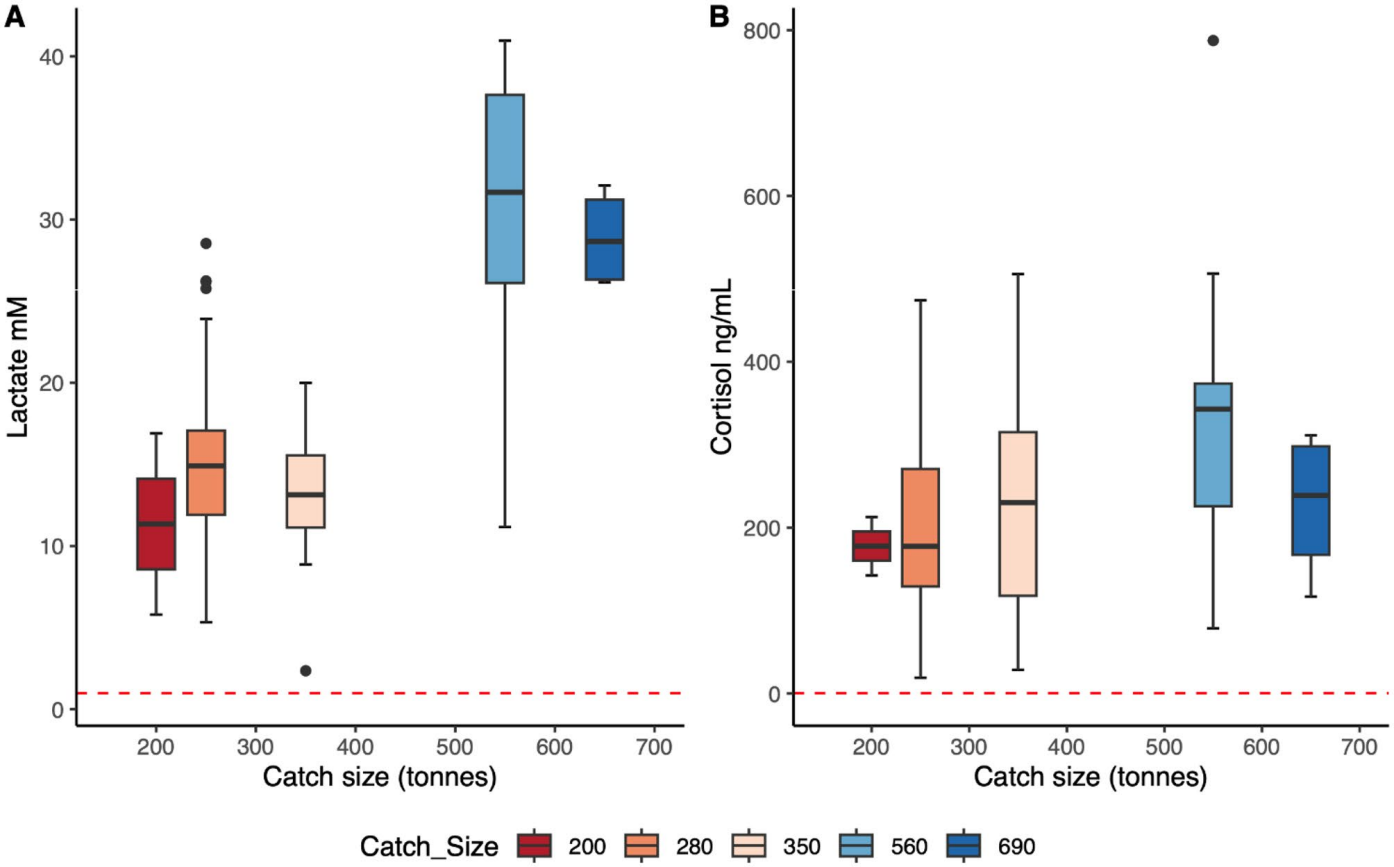
Plasma concentrations of lactate and cortisol of Atlantic herring (*Clupea harengus*) following trawling and pumping in the North Sea at five different catch sizes. Values at each catch size are presented as box-plots for lactate (A) and cortisol (B). Significant positive correlations between catch size and both lactate (p<0.001) and cortisol (p=0.004) were identified using a spearman correlation test. The relationship between catch size was strong (correlation coefficient = 0.72) for lactate while weak (correlation coefficient = 0.34) for cortisol. The dashed red lines are included for illustrative purposes only and represents the mean plasma concentrations of lactate and cortisol respectively in a group of reference fish caught with a hook and line.

No significant effects of either trawling time or catch size were found on scale loss (d.f = 3, p = 0.08171 and d.f = 2, p = 0.2123, respectively). Plasma osmolality increased with trawling time (d.f = 3, p < 0.0001) with values of 434.85 ± 36.81 mmol/kg following a 60 min trawl to 523.0 ± 49.5 mmol/kg following a 300 min trawl (Fig. 4). There was also a positive correlation between osmolality and trawling time (Spearman's rank correlation, d.f = 66, p < 0.001). No significant positive effects were found of trawling time on cortisol, lactate or chloride (d.f = 3, p = 0.06169, 0.05783 and 0.1799, respectively). Plasma levels of lactate increased significantly (d.f = 4, p < 0.0001) with catch size with values of 9.5 ± 6.4 mM at 200 tonnes to 30.6 ± 8.8 mM at 560 tonnes (Fig. 5A). A similar significant trend was observed in the plasma levels of cortisol, which increased from 177.6 ± 49.8 ng/mL at a catch size of 200 tonnes to 331.6 a ± 168.1 ng/mL at 560 tonnes (Fig. 5B). There was a positive correlation between catch size and cortisol (Spearman's rank correlation, df = 65, p-value = 0.003924) and lactate (df = 25, p-value < 0.001). No significant positive effects were found of catch size on plasma osmolality or chloride (p = 0.08563 and p = 0.2123, respectively). Also worth noting is that the highest mortalities (100%) occurred following the two shortest hauls (both 60 min long) with the two largest catches (560 and 690 tonnes, respectively).

Figures 4 and 5 only show positive significant results such as effects of trawling time on osmolality, effects of catch size on lactate and effects of catch size on cortisol.

### The relationship between blood haemolysis and moribund fish

It was evident that the vast majority (95%) of the blood sampled from herring captured with the trawl was haemolysed. Targeting live fish (*i.e*., fish that did not score 0 on all three assessed reflexes) significantly reduced (d.f = 1, p < 0.0001) the occurrence of haemolysed samples down to 10%. No significant differences between the live and haemolysed fish were found when assessing the morphological indicators or internal damage occurrence.

### The effect of time elapsed since death

No significant effects of time elapsed since death (i.e., 15, 30, 45, 60, 120 or 180 min) were found in plasma osmolality, cortisol or chloride. On average, the plasma levels were 368.2 ± 5.4 mmol/Kg, 6.1 ± 0.6 ng/ml and 139.8 ± 4.95 mmol/L for osmolality, cortisol, and chloride, respectively. Plasma lactate increased significantly (d.f = 8.747 p = 0.011) from 4.8 ± 0.6 mM at 15 min postmortem to 11.8 ± 0.7 mM at 180 min postmortem. Also worth noting is that not even at 180 min postmortem was there haemolysis present in the blood sampled from herring caught with hook and line.

## Discussion

There has been an increasing interest in the welfare of fish captured in commercial fisheries^12,13,36^. Nevertheless, little is known about how different fishing methods, routines for pumping, sorting, handling, and killing after capture, affect the health and welfare of fish. Our results show that capture with pelagic trawling and subsequent pumping onto the vessel is stressful and has a negative effect on the health and welfare of Atlantic herring. By the time fish were pumped out of the trawl and had reached the water separator of the vessel, approximately 96% of all fish that were assessed were presumed dead (ranging between 53 and 100% depending on the specific fishing haul). This largely contrasts with our reference fish, caught using hook and line, where all the sampled fish were alive following capture. Instances of high mortality rates when targeting species with active fishing gear (*e.g*. purse seines and operated trawls) have been documented for several fish species, including Atlantic herring^15,19,47,48^. Although we cannot conclusively identify the factor/s underlying mortality or when death occurs, our results indicate that the herring experience severe stress before dying, as evidenced by the investigated haematological indicators of stress (*i.e.,* plasma levels of cortisol, plasma lactate, plasma osmolality and chloride).

One suggested explanation for the high mortalities in commercial fisheries is wounding and other skin injuries^16,17^. Here, wounding did not explain the high mortalities observed as only a low prevalence (5%) of such injuries (*i.e.,* fin damage, skin lesion, opercular damage, eye damage and internal damage) were found in the Atlantic herring, and the presence of such injuries were similar in both live and dead fish. Our results proved even more interesting when accounting for the extended trawling times of the first three hauls (> 60 min), as previous studies have shown it to be one of the main reasons behind skin injuries^13^.

However, we found a high scale loss (~ 95%) following trawling, regardless of whether the individual was presumed alive or dead. Extensive scale loss will deteriorate the skin's protective barrier function and impair the fish's osmoregulatory capacity^47,49^. In a marine environment where teleost fishes, including Atlantic herring, are hypoosmotic to the surrounding seawater, the skin barrier prevents excessive sodium chloride from the seawater from entering the fish and its blood^50^.

The severe scale loss, the high plasma osmolality and chloride concentrations may alone or in combination explain the high occurrence of blood haemolysis in herring caught with the pelagic trawl^51^. The susceptibility of Atlantic herring blood to haemolysis, influenced by scale loss and resulting in high mortalities, has been previously documented in a study on the effects of descaling in Atlantic herring^47^. The reasons and consequences of the severe haemolysis found here are not yet fully understood, but it was clear that only from the few fish that showed some degree of consciousness could a blood sample with intact red blood cells be attained. Furthermore, since there were no observations of haemolysis in the reference fish, even when sampled for up to 180 min postmortem, this phenomenon is not merely a consequence of the fish being dead when the blood samples were collected.

Trawling duration and catch size are other factors suggested to affect the experienced stress and survival of fish in the trawl^21,23,24,52,53^. It is important to note that these two factors are likely not independent but interact with each other. Therefore, all interpretations of their individual effects should be approached with caution. Our results suggest that catch size rather than trawling duration was the main determining factor driving the high mortalities of herring caught using pelagic trawl. This is because the highest mortalities were observed following the two largest catches, even though the trawling duration during these two hauls was 1–2 h shorter compared to hauls with smaller catches. However, we did see a correlation between increased plasma osmolality and increased trawling times, which is likely a consequence of the increased time spent in the hyperosmotic seawater. In a stressful environment where oxygen demands are increased, there is a need for a higher osmoregulatory effort, which will result in an also higher tissue permeability. This phenomenon is known as the osmorespiratory compromise, and it becomes even more severe with the skin barrier impairment due to the massive scale loss^49^. Catch size with high densities in the cod-end (*i.e.,* the narrow end of a tapered trawl net) have been pointed out as a reason for the high mortalities associated with trawling^15,20^. When catch sizes are large, the fish are exposed to both intensive mechanical stress and hypoxia (*i.e.,* the animal is deprived of adequate oxygen supply). Also, the high frequency of scale loss found here indicates that the fish experienced mechanical stress during trawling and pumping. However, the scale loss was high in all sampled fish independent of trawling duration and catch size.

Plasma lactate has previously been used to study stress and exhaustive exercise following fishing in both herring and European pilchard (*Sardina pilchardus*)^21–23^. Here, we observed increased plasma lactate levels with increasing catch size, indicating that the fish to an increasing extent had to rely on anaerobic energy production when experiencing the more severe hypoxic conditions accumulating in a densely packed trawl^23,52^. Furthermore, it has been shown that even free-swimming pelagic species may experience significant reductions in oxygen availability when positioned in the centre of a large school^54^. In the present study, we did not have the chance to measure oxygen availability amongst the fish in the trawl, but as the density of herring in any trawl used in commercial fisheries will be magnitudes higher compared to that of a wild school^54^, it is safe to assume that the oxygen availability will be severely limited also within the trawl. However, the oxygen availability will likely not be evenly distributed throughout the trawl as fish on the outer rim of the trawl will be less affected by hypoxia than fish in the centre. Also, the severity of the hypoxia increases when the vessel stops, and the cod-end is connected to the fish pump as the trawl is no longer dragged through aerated seawater. This, together with the variation in the time elapsed since different individual fish entered the trawl, most likely explains the high variation observed in the different investigated haematological variables. Such confounding factors are largely inevitable in this kind of field study.

Although no significant differences in plasma cortisol levels were detected in relation to catch size and trawling times, it is important to note that cortisol concentrations, in our study, varied over time. This variation is understandable, given the uncertainty regarding the exact time each fish enters the net. Fish entering the net shortly before the end of trawling are expected to exhibit different physiological levels compared to those that have been swimming in the net for an extended period. Additionally, it is highly probable that the mechanical stress associated with trawling and pumping may be excessive for the individuals to endure, rendering them incapable of producing additional cortisol.

Taken together, our results show that capture with pelagic trawl and pumping onto the vessel had negative effects on the welfare of Atlantic Herring and highlight the importance of the ongoing developments of fishing gears and routines that are gentler on the fish.

## Conclusion

From our results, it is not possible to pinpoint the timing and primary cause of death for the trawl caught Atlantic Herring. Nevertheless, the high mortalities, excessive scale loss, haemolysis and pronounced stress responses observed indicate that Atlantic Herring is a very sensitive species and that the investigated method of capture negatively affected their welfare. Today, efforts are being made to develop effective on-board stunners and methods to actively kill the fish, to be used on wild-caught fish as a way to improve both fish welfare and fillet quality^55,56^. Notably, though, our findings highlight that at least for RSW vessels focusing on large catch sizes of Atlantic Herring using pelagic trawls, future research and development efforts should instead focus on improving fish welfare within the trawl and when pumped as the vast majority of fish are already dead when they come on-board. However, with the enormous diversity seen in commercial fisheries (*i.e.* in terms of targeted species, fishing methods, catch sizes, etc*.*), with reports of close to 100% survival of their catches^57,58^, developing effective on-board stunners on these vessels would still be of great importance. Protecting fish welfare during capture and killing is about minimising the pain, distress or suffering of the animals. This is achieved by minimising both the severity and duration of the distress and pain the fish are subjected to. Therefore, future studies should aim at trying to determine when the fish dies during the fishing procedure and the severity of the stress they have experienced before they do so. Future studies should also investigate the underlying reasons and the consequences of the haemolysis of red blood cells on the quality of the flesh. It is known that haemolysis accelerates lipid oxidation in the fish muscles, giving rise to unwanted flavours, pigmentation, textural changes, and nutritional loss^59–61^. Consequently, if improvements are made that will safeguard the fish’s welfare and limit haemolysis, this can also positively affect the quality and, hence, the value of the fish. Nevertheless, it is important to note that more and more time has been spent on commercial fisheries welfare research. Previous studies on capture and release, behaviour, and physiological responses^13,17,22,28,36,42,48,56^ have given new insight on how to improve the welfare of wild-caught fish.

### Supplementary Information

Supplementary Information.

## Data Availability

The datasets generated and/or analysed during the current study are available in the figshare repository, found in: https://figshare.com/projects/Investigating_the_effects_of_pelagic_trawling_on_the_welfare_of_Atlantic_herring_Clupea_harengus_/201,963. https://figshare.com/projects/Investigating_the_effects_of_pelagic_trawling_on_the_welfare_of_Atlantic_herring_Clupea_harengus_/201963.
