# Supplementary material for: Investigating the effects of pelagic trawling on the welfare of Atlantic herring (Clupea harengus)
Source: Sci Rep. 2024 Jul 30;14:17530. doi: 10.1038/s41598-024-68629-8 (PMC11289377; doi:10.1038/s41598-024-68629-8)
Supplement: Supplementary file 1 — Supplementary Information. [file 41598_2024_68629_MOESM1_ESM.pptx]

## Slide 1
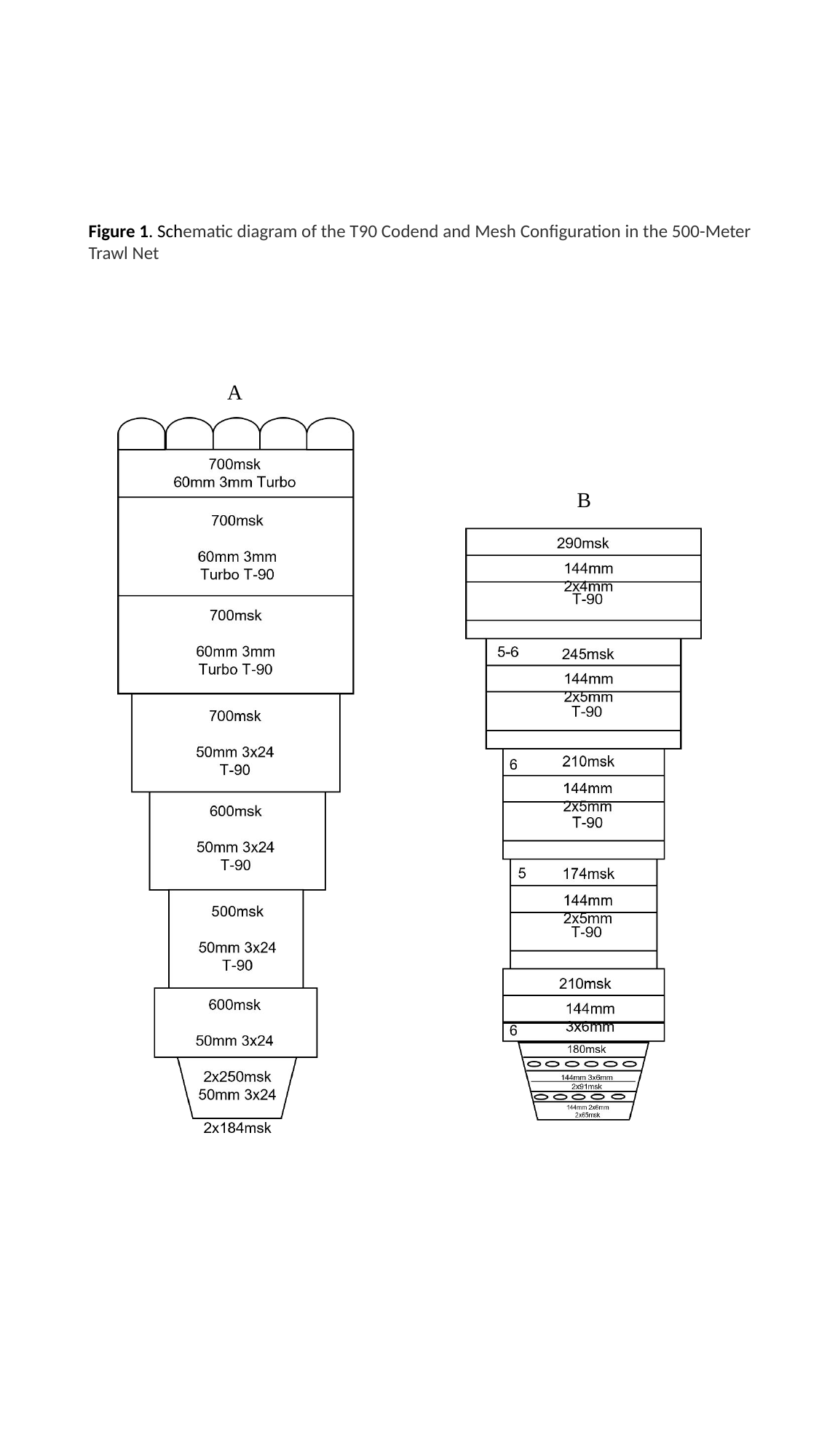

Figure 1. Schematic diagram of the T90 Codend and Mesh Configuration in the 500-Meter Trawl Net
A
B

## Slide 2
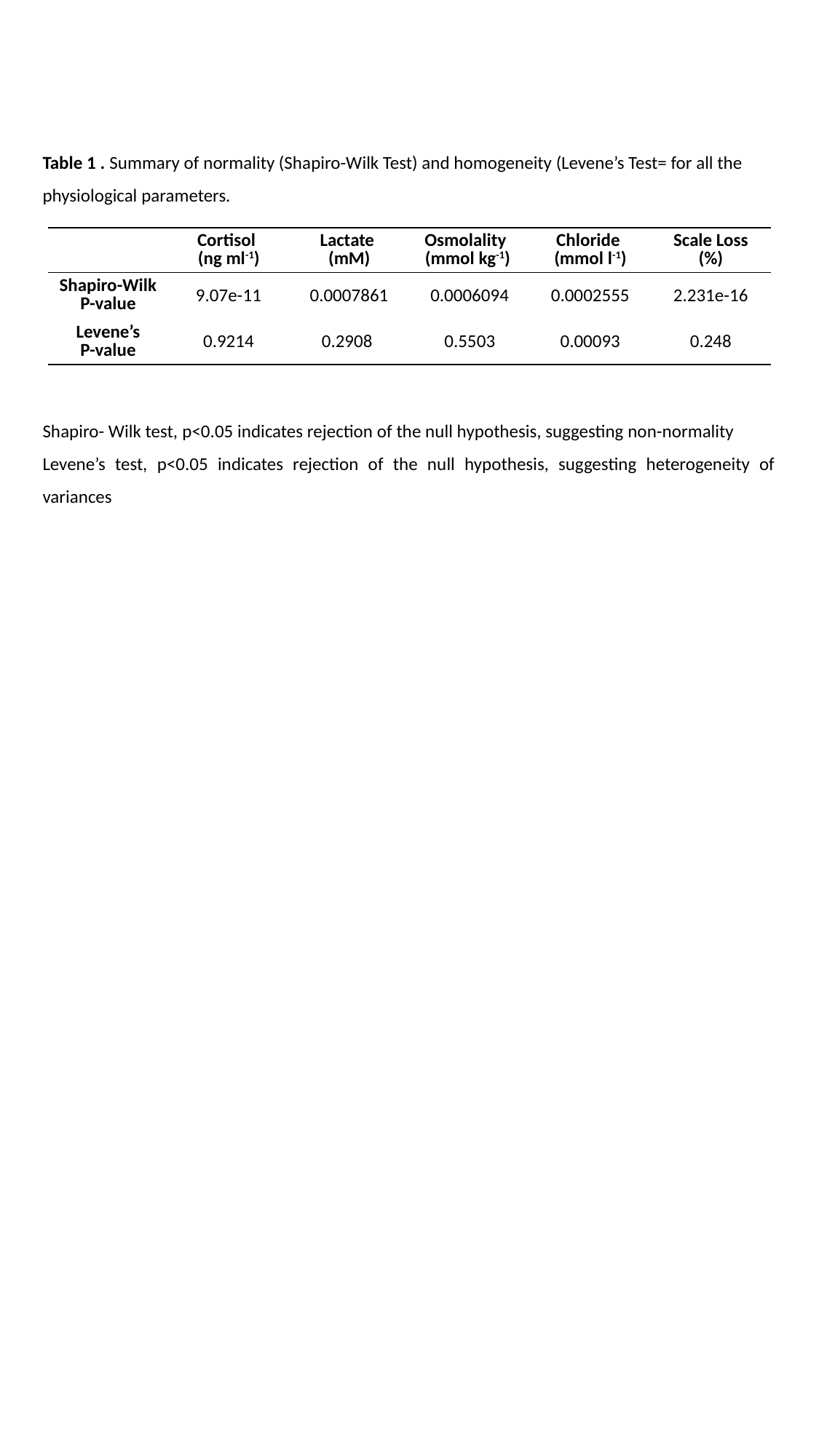

Table 1 . Summary of normality (Shapiro-Wilk Test) and homogeneity (Levene’s Test= for all the physiological parameters.
| | Cortisol (ng ml-1) | Lactate (mM) | Osmolality (mmol kg-1) | Chloride (mmol l-1) | Scale Loss (%) |
| --- | --- | --- | --- | --- | --- |
| Shapiro-Wilk P-value | 9.07e-11 | 0.0007861 | 0.0006094 | 0.0002555 | 2.231e-16 |
| Levene’s P-value | 0.9214 | 0.2908 | 0.5503 | 0.00093 | 0.248 |
Shapiro- Wilk test, p<0.05 indicates rejection of the null hypothesis, suggesting non-normality
Levene’s test, p<0.05 indicates rejection of the null hypothesis, suggesting heterogeneity of variances
